# Structure, phase transition and properties of the one-dimensional antiferromagnet Cu(2,6-dimethylpyrazine)Br_2_†

**DOI:** 10.1039/d1ra02318g

**Published:** 2021-06-25

**Authors:** Fei Ding, Chuanlu Yang, Xiangnan Gong, Hui Zheng, Xiaoyu Zhou, Lingli Li, Lichun Zhang, Dehua Wang, Bingying Pan

**Affiliations:** School of Physics and Optoelectronic Engineering, Ludong University Yantai Shandong 264025 China bypan@ldu.edu.cn; Analytical and Testing Center, Chongqing University Chongqing 401331 China; School of Chemistry and Materials Science, Ludong University Yantai Shandong 264025 China

## Abstract

We report the crystal structure and properties of the one-dimensional *S* = 1/2 antiferromagnet Cu(2,6-dimethylpyrazine)Br_2_ with strong intra-chain exchange. At room temperature, its linear spin chains are formed by Cu^2+^ ions *via* the non-bonding Br⋯Br contacts. Interestingly, a phase transition from *Pmmn* to *P*21/*n* structure occurs at *T*_S_ ≈ 248 K below which the [CuBr_2_]_*n*_ spin chains become non-linear and the magnetic susceptibility abruptly increases, reflecting the weakening of antiferromagnetic exchange strength. This result evidences the Goodenough–Kanamori rules which claim that a linear super-exchange pathway produces stronger antiferromagnetic coupling. From magnetic susceptibility measurements we find the average intra-chain exchange strength is *J*/*k*_B_ ≈ −88.18 K in the low temperature phase. Both magnetic and specific measurements show the absence of magnetic ordering down to 2 K, implying the excellent magnetic one-dimensionality of Cu(2,6-dimethylpyrazine)Br_2_. We also performed ultra-violet (UV) absorption and photoluminescence measurements which give a semiconducting band gap *Δ* ≈ 2.79 eV which is consistent with theoretical calculations.

## Abstract 2

Novel transition from linear to non-linear chain structure was discovered in a one-dimensional magnet Cu(2,6-dimethylpyrazine)Br_2_.

## Graphical abstract

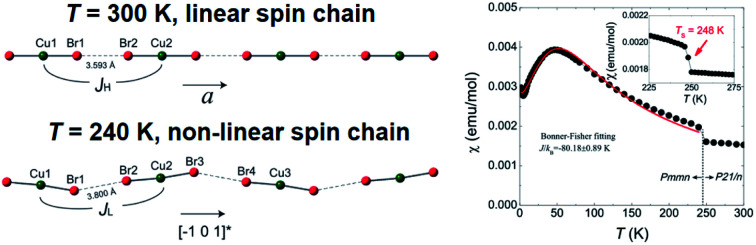

## Introduction

The synthesis and physical properties of coordination polymer complexes with the chemical formula LCuX_2_ (L = organic ligand, X = halide ions) is an important subject of inorganic chemistry, because these materials play a crucial role for understanding the pathway of magnetic interactions. Especially, for the quantum antiferromagnets, the superexchange pathways are highly relevant to the specific structure of the compound.^1–3^ The Cu^2+^ ion with one unpaired electron in coordination polymers exhibits the smallest spin number (*S* = 1/2) and strongest quantum fluctuations, and thus is widely used to study quantum magnetism. Depending on the organic ligand species and coordination environment of the Cu^2+^ ions, various superexchange pathways and magnetic behaviors can form in different coordination polymers.^3^

One-dimensional (1D) magnetic systems have been continuously drawing research interests for their novel quantum states, excitations, and strong quantum fluctuations.^4^ In the past decades, various copper(ii) coordination complexes have been reported as material realizations of 1D quantum spin models.^1–3^ The properties of 1D magnets are directly affected by the superexchange pathways which are dependent on the concrete crystalline structure. Especially, the non-bonding contacts between halide ions (Cu–X ⋯X–Cu, X = Br, Cl) represents an important type of superexchange pathways and have been widely studied.^1,3^ The sign and strength of the magnetic interaction *via* such a two-halide bridge depend on various parameters such as the geometry of the chains and the distance of the bridging halide ions.^1,5^ In fact, all related variables in the coordination environment have potential influence on the properties of 1D magnetism.^1^ For example, studies show that the Br⋯Br contacts propagate stronger exchanges than their Cl⋯Cl counterparts in all cases.^6–8^ Upon to date, a full description on the magnetic interaction *via* the two-halide bridge is still lacking.

The pyrazine-based compound Cu(pyrazine)(NO_3_)_2_ is a model 1D Heisenberg antiferromagnet and has been extensively studied by neutron scattering,^9^ muon-spin relaxation (μSR),^10^ nuclear magnetic resonance (NMR) spectroscopy,^11,12^ and thermal transport.^13^ Substituting pyrazine with related ligands such as phenazine, quinoxaline, methylpyrazine, and chloropyrazine also result in a variety of linear spin chains, but the magnetic super-exchange through pyrazine ring is weak with *J*/*k*_B_ smaller than −10.3 K.^14^ By using Br as anions, a derivative 1D antiferromagnet Cu(2,5-dimethylpyrazine)Br_2_ exhibit a exceptionally strong exchange with *J*/*k*_B_ = −234 K and the authors demonstrate the dominate antiferromagnetic exchange propagate through the non-bonding Cu–Br ⋯Br–Cu pathway.^15^ Such strong exchange is also very unusual among the 1D magnets mediated by the two bromide contacts.^1^ It is of great interest to investigate related compounds in order to explore the mechanism of enhanced superexchange in this two-halide bridged spin chain system.

Cu(2,6-dimethylpyrazine)Br_2_ (1) is an analog compound of Cu(2,5-dimethylpyrazine)Br_2_ but its structure has not been reported in literature. The preliminary magnetic susceptibility of 1 was reported by Inman *et al.* in 1972 on powder sample and the data was explained by an antiferromagnetic spin chain model with *J* = −47.5 K.^2^ However, no magnetic susceptibility data is available in a wide temperature range above 100 K, leaving large uncertainty on the data fitting result.^2^

In this work, we report the structure, phase transition, magnetism, specific heat and optical properties of 1. At room temperature, 1 is featured by [CuBr_2_]_*n*_ spin chains, similar to Cu(2,5-dimethylpyrazine)Br_2_. Surprisingly, an unique phase transition from *Pmmn* to *P*21/*n* structure occurs at *T*_S_ = 248 K, resulting in significant deformation of the spin chains at the low temperature (LT) phase. This structural transition is accompanied by a sharp jump in magnetic susceptibility across *T*_S_, indicative of the weakening of the intra-chain exchange strength once entering the LT phase. By fitting the LT susceptibility data by a uniform spin chain model we find the average exchange strength is −88.18 K, this strength is comparable to that of Cu(2,5-dimethylpyrazine)Br_2_. The specific heat data do not show any feature of magnetic ordering down to 2 K, indicating excellent 1D magnetism of 1. Our work reveals 1 is an unique 1D system that shows linear to non-linear transition in spin chains which has not been found in other copper(ii) coordination complexes at ambient pressure. This system thus provides a novel platform to study the mechanism of spin chain geometry on the two-halide bridge mediated magnetic superexchange.

## Experimental

### Synthesis

Single crystals of Cu(2,6-dimethylpyrazine)Br_2_ were synthesized by conventional diffusion method. 4 mmol 2,6-methylpyrazine (>99%, Adamas-beta) and 4 mmol copper bromide (>99%, Adamas-beta) were loaded into the 250 mL and 50 mL beakers, respectively. The 50 mL beaker was put inside the larger one and alcohol was slowly introduced till the alcohol level is far up beyond the edge of the 50 mL beaker. After about one week, black single crystals were formed. The typical single crystal size is 3 mm × 0.25 mm × 0.1 mm. Anal. calc.: C, 21.72%; H, 2.41%; N, 8.45%; found: C, 21.76%; H, 2.62%; N, 8.44%.

### X-ray diffraction

We used the three-circle diffractometer (Mo K_α_ radiation, *λ* = 0.71073 Å) for the X-ray diffraction of Cu(2,6-dimethylpyrazine)Br_2_ single crystal. High quality diffraction data were collected at 300 and 240 K and the crystal structures were solved by the direct method and refined *via* full-matrix least-square techniques using the Olex2 and SHELXL program package.^16,17^ The resulted cell parameters, selective atomic coordinates, bond lengths and bond angles are listed in Tables 1 and 2. Crystallographic data of Cu(2,6-dimethylpyrazine)Br_2_ have been deposited at the Cambridge Crystallographic Data Center (CCDC No. 2070208 (*T* = 300 K) and 2070209 (*T* = 240 K)).

**Table 1:** Crystal data and structure refinement of Cu(2,6-dimethylpyrazine)Br_2_ at 300 and 240 K

Temperature (K)	300(2)	240(2)
Empirical formula	(C_6_H_8_N_2_)CuBr_2_	(C_6_H_8_N_2_)CuBr_2_
Formula weight	331.50	331.50
Space group	*Pmmn*	*P*21/*n*
Crystal system	Orthorhombic	Monoclinic
Wavelength	0.71073 Å	0.71073 Å
Lattice parameters	*a* = 8.3552(9) Å	a* = 10.2576(5) Å
	*b* = 8.5782(11) Å	b* = 8.3613(5) Å
	*c* = 6.6959(7) Å	c* = 11.2178(5) Å
	*α* = *β* = *γ* = 90°	α* = γ* = 90°
		β* = 102.982(2)°
*Z*	2	4
*μ* (mm^−1^)	10.541	10.791
*θ*_min_/*θ*_max_ (°)	6.808/77.732	4.866/70.382
Independent reflections	1468	4262
*R*_int_	0.0552	0.0649
*F*(000)	314.0	628.0
Restraints/parameters	0/37	0/105
Goodness-of-fit on *F*^2^	1.023	1.051
*R*_1_/w*R*_2_ [*I* ≥ 2*σ*(*I*)]	0.0395/0.0795	0.0551/0.1340
Largest diff. peak/hole (e Å^−3^)	1.39/−0.97	1.46/−1.30

**Table 2:** Bond lengths and angles for the [CuBr_2_]_*n*_ magnetic chains of 1 at 300 K and 240 K

	300 K	240 K
**Bond lengths**		
Cu1–Br1	2.3813(5) Å	2.37(1) Å
Br1–Br2	3.593(2) Å	3.80(1) Å
Cu2–Br2	2.3813(5) Å	2.38(6) Å
Cu2–Br3	2.3813(5) Å	2.37(1) Å
Br3–Br4	3.593(2) Å	3.80(1) Å
Cu3–Br4	2.3813(5) Å	2.38(6) Å

**Bond angles**		
Cu1–Br1–Br2	180.0°	156.51(2)°
Br1–Br2–Cu2	179.84(9)°	167.20(6)°
Br2–Cu2–Br3	179.69(4)°	175.09(9)°
Cu2–Br3–Br4	180.0°	156.51(1)°
Br3–Br4–Cu3	179.84(9)°	167.20(6)°

### Elemental analysis, photoluminescence, and UV absorption measurements

Elemental analysis were determined on an Vario EL cube analyzer. Photoluminescence (PL) measurements were conducted with a 325 nm He-Cd laser as the pump source (Kimmon Electric, Ltd., Saitama, Japan) and a monochromator (SR-500i-A, Andor Technology, Belfast, UK) working at room temperature. The characteristic absorption peak was measured by a Shimadzu UV-2550 spectrophotometer.

### Magnetic susceptibility and specific heat measurements

The magnetic susceptibility properties were measured by a MPMS SQUID magnetometer (Quantum Design) with magnetic field up to 7 T. The measured temperature range is from 300 to 2 K. Specific heat was measured by a Physical Property Measurement System (PPMS, Quantum Design) down to 2 K.

## Results and discussion

**Fig. 1:**
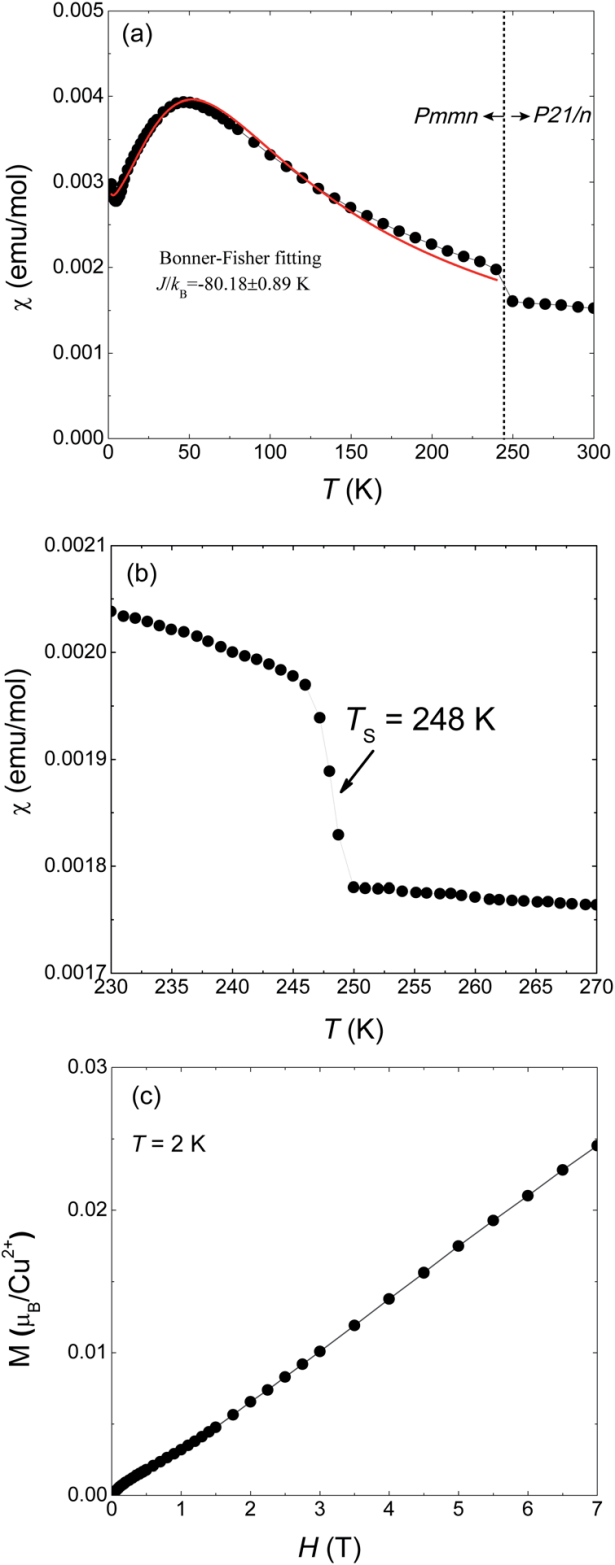
Magnetic susceptibility and magnetization of Cu(2,6-dimethylpyrazine)Br_2_ powdered sample. (a) Magnetic susceptibility in temperature range 2–300 K under an applied field of *H* = 1000 Oe. (b) The jump of *χ* at *T*_S_ = 248 K corresponds to the orthorhombic-to-monoclinic structure phase transition. (c) Magnetization data as a function of field up to 7 T at *T* = 2 K.

Magnetic susceptibility of 1 on powder sample was measured under 1000 Oe from 300 to 2 K (Fig. 1(a)). A abrupt jump in *χ* appears around *T*_S_ = 248 K which corresponds to a phase transition from high temperature (HT) phase to a low temperature (LT) phase. Fig. 1(b) is a detailed measurement around *T*_S_. Such a discontinuous jump at *T*_S_ can not be attributed to a second-order transition but arises from a first-order structural transition, as also evidenced by our X-ray diffraction results which will be shown later. At lower temperatures a maximum appears at around 50 K and then decreases, a typical behavior for one-dimensional magnets. The maximum temperature is consistent with the results of Inman *et al.* which is 47 K.^2^ We used the Bonner–Fisher formula for a Heisenberg magnetic chain to approximately describe the magnetic susceptibility of the LT phase.^18^ The fitting is represented by the red solid line in Fig. 1(a) which gives *J*_L_/*k*_B_ = −88.18 ± 0.89 K and *g* = 2.40 ± 0.01.

The magnetization at 2 K as a function of applied field is shown in Fig. 1(c) in which *M* varies quasi-linearly with *H* up to 7 T. Especially, the measured magnetization at 7 T is only 0.25 *μ*_B_/Cu^2+^, far smaller than the spin-only saturated value 1.73 *μ*_B_/Cu^2+^ of Cu^2+^ with spin −1/2. This behavior again indicates strong antiferromagnetic coupling in 1.

**Fig. 2:**
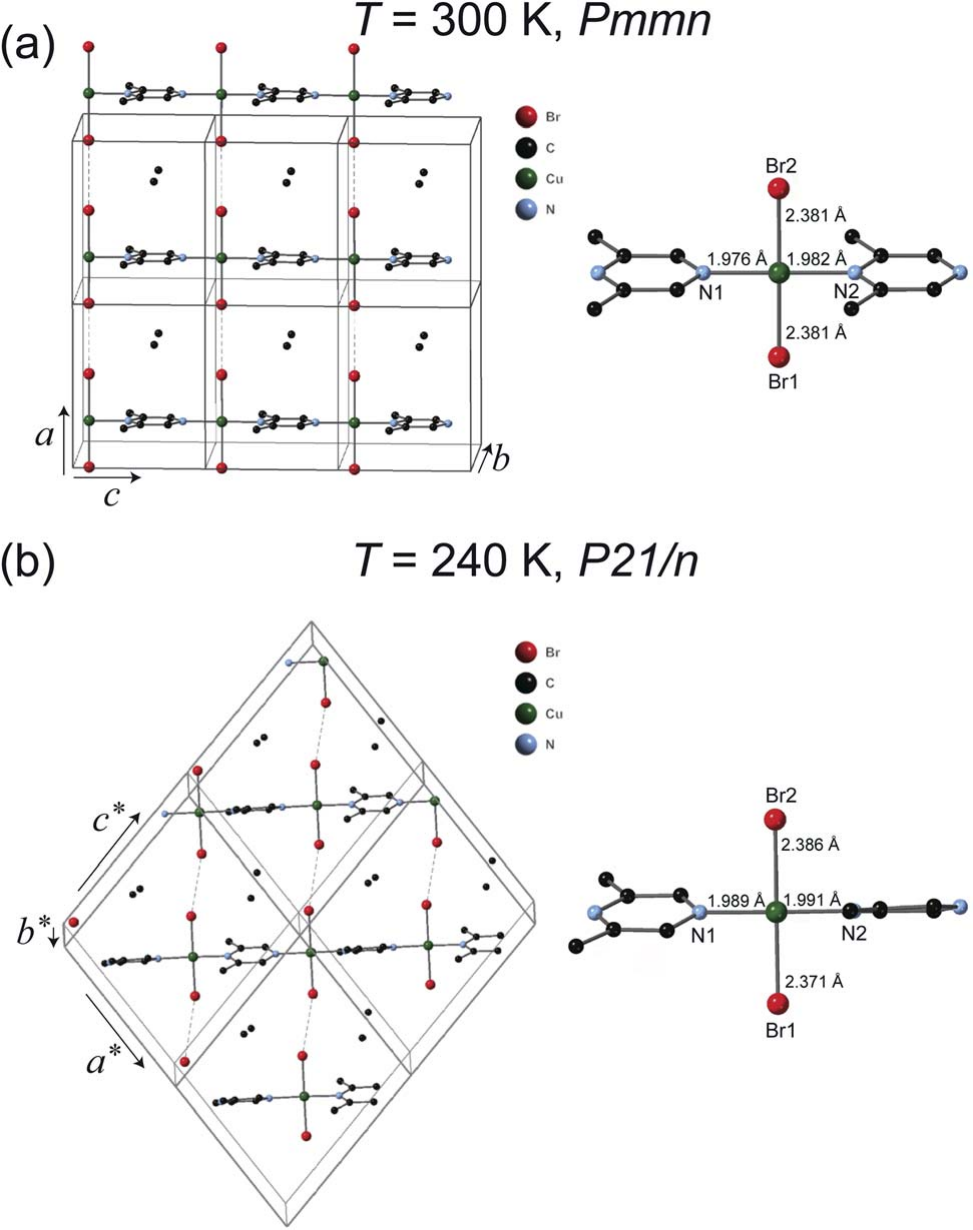
Crystal structures of 1. (a) The high temperature (HT) orthorhombic structure phase (*T* = 300 K). (b) The low temperature (LT) monoclinic structure phase (*T* = 240 K). The coordination environment of copper(ii) is shown in the right panel. Hydrogen atoms are omitted for clarity.

The crystal structures of 1 were solved at 300 K and 240 K. The refinement results are listed in Table 1. The chemical formula of 1 is (C_6_H_8_N_2_)CuBr_2_ which can also be verify by the thermogravimetric analysis measurement (see ESI†) and elemental analysis. As can be seen in Fig. 2(a), at 300 K the space group of 1 is *Pmmn* with the lattice parameters *a* = 8.3552(9) Å, *b* = 8.5782(11) Å, and *c* = 6.6959(7) Å. The structure of 1 is similar to its analogous coordination compound Cu(2,5-dimethylpyrazine)Br_2_ (ref. 15) with –Cu–2,6-dimethylpyrazine–Cu– infinite structure along the *c* axis. The copper(ii) atom is tetrahedrally coordinated by two bromide atoms and two N atom from the 2,5-dimethylpyrazine ring. The Cu–Br bond length is 2.381 Å, and the Cu–N bond lengths are 1.976–1.982 Å (right panel of Fig. 2(a)). The linear Cu–Br⋯Cu magnetic chains propagate along the *a* axis with the non-bonding two-halide contacts (Br⋯Br length *d*_Br⋯Br_ = 3.593 Å, Cu–Br⋯Br angle = 180.0°). The mean plane of 2,6-dimethylpyrazine ring perpendicular to the chain direction. In view that *d*_Br⋯Br_ = 3.632 Å in Cu(2,5-dimethylpyrazine)Br_2_,^15^ both compounds should also have similar intra-chain super-exchange strength.

**Fig. 3:**
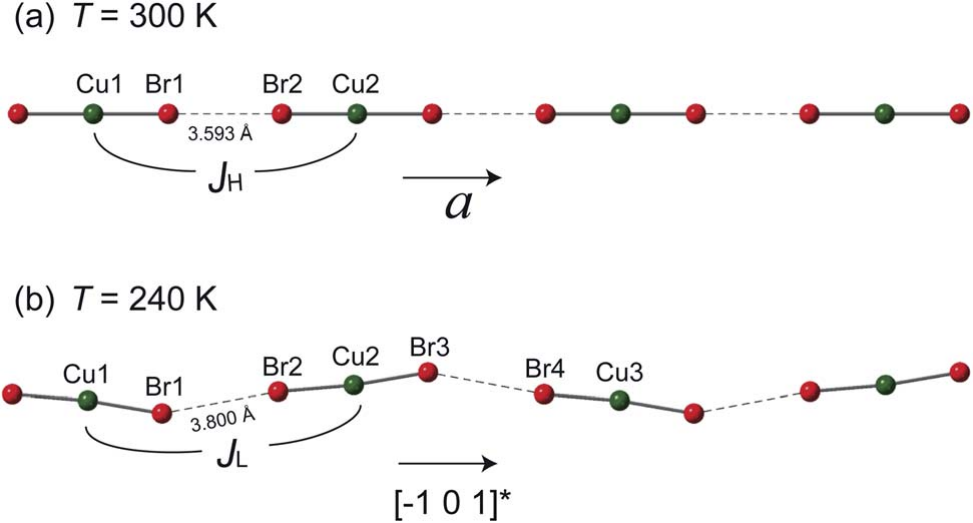
The [CuBr_2_]_*n*_ magnetic chains at (a) 300 K and (b) 240 K. The antiferromagnetic super-exchanges between neighboring copper ions through the Br⋯Br non-bonding contact are denoted as *J*_H_ and *J*_L_ for the HT and LT phases, respectively. The detailed bond lengths and bond angles of the spin chains are listed in Table 2.

However, the structure of 1 at 240 K dramatically changes to the monoclinic type with *P*21/*n* structure. At the LT phase, the Cu–N and Cu–Br2 bond lengths were enlarged to 1.989–1.991 and 2.386 Å, respectively. Only the Cu–Br1 bond length was shortened to 2.371 Å. The 2,6-dimethylpyrazine rings also display rotation around the N1–Cu–N2 axis (right panel of Fig. 2(b)). The two-halide contacts are obviously distorted to form a non-linear spin chain (Br⋯Br length *d*_Br⋯Br_ = 3.80(1) Å, Cu–Br⋯Br angle = 156.51(2)°). These large lattice distortions across *T*_S_ make the [CuBr_2_]_*n*_ magnetic chains substantially twisted, rendering the crystal to a lower symmetry. The [CuBr_2_]_*n*_ chain structure and its detailed length/angle parameters at 300 and 240 K are shown in Fig. 3 and Table 2.

The observation of structural phase transition in 1 is surprising because it has never been found in its analog compounds like Cu(2,5-dimethylpyrazine)Br_2_, Cu(2,6-dimethylpyrazine)(NO_3_)_2_, or other pyrazine-based coordination complexes at ambient pressure.^2,15,19–21^ The linear to non-linear structural transition of [CuBr_2_]_*n*_ chains bring substantial change in the coordination environment and should have considerable impact on its 1D magnetism.

It is known that superexchange through the pyrazine ring pathway arises from the weak coupling π mechanism and the strength is less than *J*/*k*_B_ = −10.3 K is all cases.^14^ For example, the 2,6-dimethylpyrazine bridged exchange strength in Cu(2,6-dimethylpyrazine)(NO_3_)_2_ is only *J* = −4.0 ± 0.1 K,^14^ far smaller than the exchange strength in 1. So the dominate exchange in 1 through the –Cu–2,6-dimethylpyrazine–Cu– pathway is unlikely. Butcher *et al.* investigated the structure and magnetic behavior of Cu(2,5-dimethylpyrazine)Br_2_ combined with quantum Monte Carlo simulations.^15^ They found the strong antiferromagnetic exchange *J* = −234 K is through the Cu–Br⋯Br–Cu pathway, whereas the Cu–2,5-dimethylpyrazine–Cu pathway only generates a much weaker strength *J*′ = −12 K. In sight its similar structure to 1, it is appropriate to attribute the propagation of *J*_L_ to the Cu–Br⋯Br–Cu pathway.

The buckling of [CuBr_2_]_*n*_ spin chain in 1 across *T*_S_ offers an unprecedented opportunity to investigate the relation of chain geometry with 1D magnetism. In the HT phase, the closest Br⋯Br is 3.593 Å which is very short in comparison with the chain structure in strong coupled tetrabromocuprate compounds.^1,22^ Together with the linear chain structure, the Cu–Br⋯Br–Cu pathway is expected to propagate strong antiferromagnetic exchange. At the LT phase, the Cu–Br⋯Br angle and the Cu–Br⋯Br–Cu dihedral angle all significantly deviate from 180°. According to the Goodenough–Kanamori rules, non-linear pathway propagate weaker superexchange.^23^ Also, the Br⋯Br contact distance is enlarged to 3.800 Å, this would further reduce the intra-chain exchange strength.^1^ The analysis is evidenced by the jump at *T*_S_ in Fig. 1(a). However, a qualitative theory should consider all geometric variables in the chain structure which is not currently available.

**Fig. 4:**
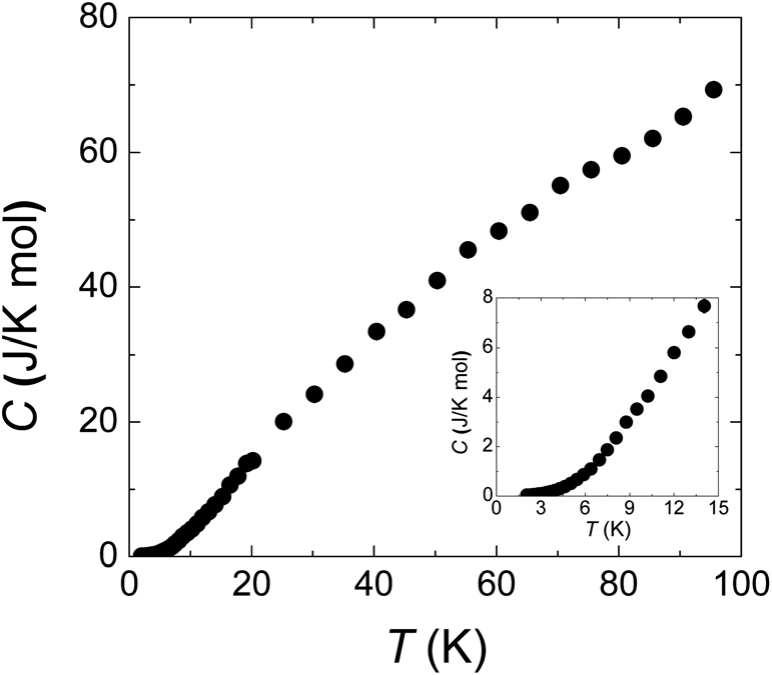
Specific heat from 2 to 100 K. The inset panel shows the data below 15 K. The smooth curve indicates the absence of long range spin ordering down to 2 K.

Fig. 4 is the specific heat data from 100 to 2 K at zero field. *C* monotonously decreases with lowering temperature. The absence of any anomaly in the measured temperature range verifies the broad peak around 50 K in *χ* (Fig. 1(a)) is purely from one-dimensional magnetism, not magnetic ordering. There is also no sign of magnetic ordering transition down to 2 K in specific heat data (inset of Fig. 4).

**Fig. 5:**
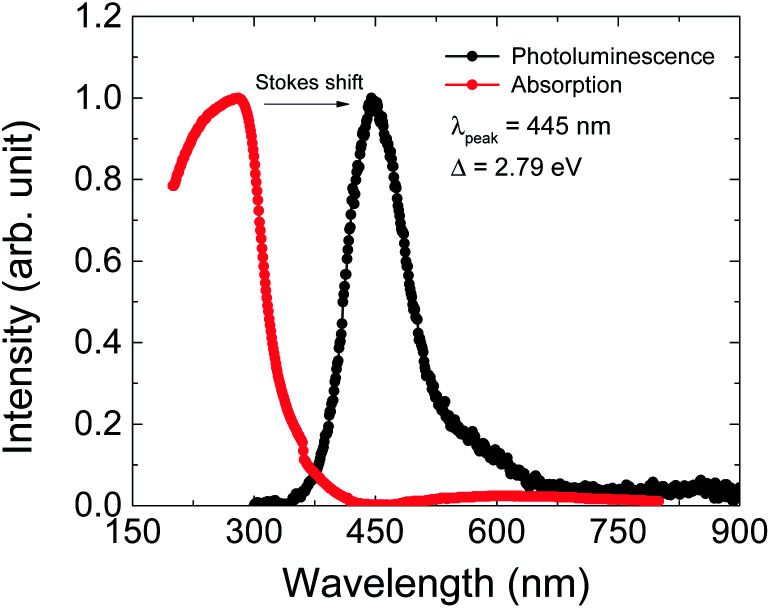
Photoluminescence (PL, black dots) and UV absorption (red dots) spectra of Cu(2,6-dimethylpyrazine)Br_2_ at room temperature. The excitation wavelength for PL is 325 nm and a strong peak appears at 445 nm, corresponding to an energy gap of *Δ* = 2.79 eV.

**Fig. 6:**
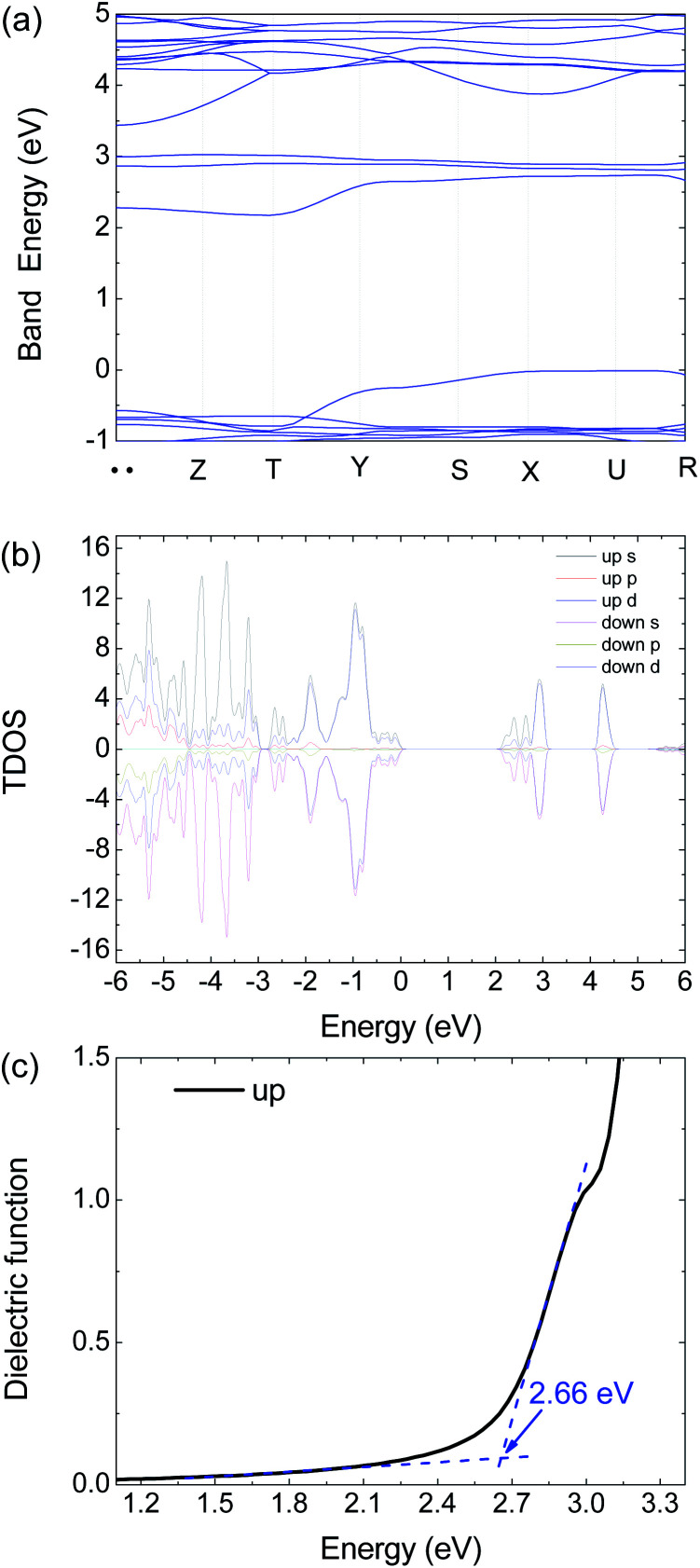
Calculated (a) electronic band structure, (b) TDOS and (c) dielectric function of 1 by HSE06 with an antiferromagnetism model.

The optical properties were investigated by PL and UV-vis absorption spectra measurements (Fig. 5). The PL spectrum was excited by a 325 nm laser source and the maximum wavelength is at 445 nm, as shown by the black dots in Fig. 6. This PL peak wavelength corresponds to an electron band gap of 2.79 eV. The PL emission peak of 445 nm can be assigned to the π–π* transition of between the organic ligand. The UV-vis absorption spectrum shows an obvious Stokes shift effect with a maximum appearing at around 280 nm.

To get further insight on its electric and optical properties, we theoretically calculated the electronic structure and optical properties of 1 by using the Vienna *ab initio* simulation package (VASP) 6.1.2 code with a projector-augmented-wave pseudopotential.^24–26^ The exchange–correlation functional for describing the electron interactions is the generalized gradient approximation with Perdew–Burke–Ernzerh of parametrization (GGA-PBE).^27^ The Heyd–Scuseria–Ernzerhof (HSE06) hybrid functional^28^ is adopted to calculate the electronic properties, optical properties, and mobility calculations because of the GGA-PBE functional usually underestimate the band energy gap.

There are 38 atoms in the unit cell with the *Pnmn* structure. The 3 × 3 × 3 Γ-centered *k*-mesh of Brillouin zone (BZ) are employed in integration, but the band structure are calculated with additional 7 special *k* points including 71 points. The energy cutoff the plane-wave basis sets is 500 eV to ensure the calculations to reach convergence. The energy convergent criterion is 10^−6^ eV. In the relaxation calculation, all the force is smaller than 0.01 eV Å^−1^ for the equilibrium structures.

The optical absorption coefficient *α*(*ω*) is used to quantitatively describe the response-ability of 1. *α*(*ω*) can be determined by the imaginary part *ε*_2_(*ω*) of the complex dielectric function *ε*(*ω*) = *ε*_1_(*ω*) + i*ε*_2_(*ω*). In the present work *ε*_2_(*ω*) is calculated by the following equation^29–31^1
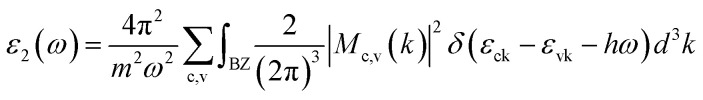
where |*M*_c,v_(*k*)|^2^ is the momentum matrix elements. The conduction and valence band states are represented by c and v. While *α*(*ω*) *via* the following expression2
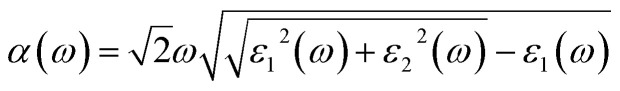
where the real part *ε*_1_(*ω*) is calculated from the imaginary part *ε*_2_(*ω*) of the complex dielectric function by using the Kramer–Kronig relationship. *ε*_2_(*ω*) is calculated by using the HSE06 functional. The calculated band structure and total density of states (TDOS) with obital characteristics of 1 are presented in Fig. 6(a and b). The calculated electronic band gap can be estimated from the dielectric function curve as presented in Fig. 6(c) which is 2.66 eV, in good agreement with the experimental value 2.79 eV from our PL measurement.

## Conclusions

In summary, we synthesized single crystalline Cu(2,6-dimethylpyrazine)Br_2_ and successively solved its structure. The linear [CuBr_2_]_*n*_ chains demonstrate strong one-dimensional antiferromagnetism *via* the two-halide exchange pathway, similar to its analog compound Cu(2,5-dimethylpyrazine)Br_2_. However, Cu(2,6-dimethylpyrazine)Br_2_ experiences a special phase transition from *Pmmn* to *P*21/*n* structure at *T*_S_ = 248 K. The [CuBr_2_]_*n*_ spin chains are non-linear and the average antiferromagnetic exchange strength get weakened after transition to the low temperature phase. Thus our experiments reveal a novel one-dimensional antiferromagnet with linear to non-linear structural transition in the spin chains and provide an example compound in the study of two-halide exchange mechanism.

## Conflicts of interest

There are no conflicts to declare.

## Supplementary Material

RA-011-D1RA02318G-s001

RA-011-D1RA02318G-s002
