# Supplementary material for: Structure, phase transition and properties of the one-dimensional antiferromagnet Cu(2,6-dimethylpyrazine)Br2
Source: RSC Adv. 2021 Jun 25;11(37):22565–70. doi: 10.1039/d1ra02318g (PMC9034352; doi:10.1039/d1ra02318g)
Supplement: RA-011-D1RA02318G-s001 [file RA-011-D1RA02318G-s001.pdf]

Electronic Supplementary Material (ESI) for RSC Advances.
This journal is © The Royal Society of Chemistry 2021

Supplementary Information

Structure, phase transition and properties of the one-dimensional antiferromagnet Cu(2,6-dimethylpyrazine)Br_2

Fei Ding[1], Chuanlu Yang[1], Xiangnan Gong[2], Hui Zheng[2], Xiaoyu Zhou[1], Lingli Li[1], Lichun Zhang[1], Dehua Wang[1] and Bingying Pan*[1]

[1] School of Physics and Optoelectronic Engineering, Ludong University, Yantai, Shandong 264025, China

[2] Analytical and Testing Center, Chongqing University, Chongqing, 401331, China

[3] School of Chemistry and Materials Science, Ludong University, Yantai, Shandong 264025, China

*Corresponding email: bypan@ldu.edu.cn

1. Thermogravimetric analysis (TGA) data

To verify the constitution and purity of **1**, we have performed thermogravimetric Analysis (TGA) was performed by NETZSCH STA 409 PC/PG instrument in N_2 flow, the hating rate is 10 °C/min. The result is shown in Fig. S1. The first step corresponds to the decomposition of 2,6-dimethylpyrazine organic ligand (calculated: 32.62\%, found: 34.51\%)

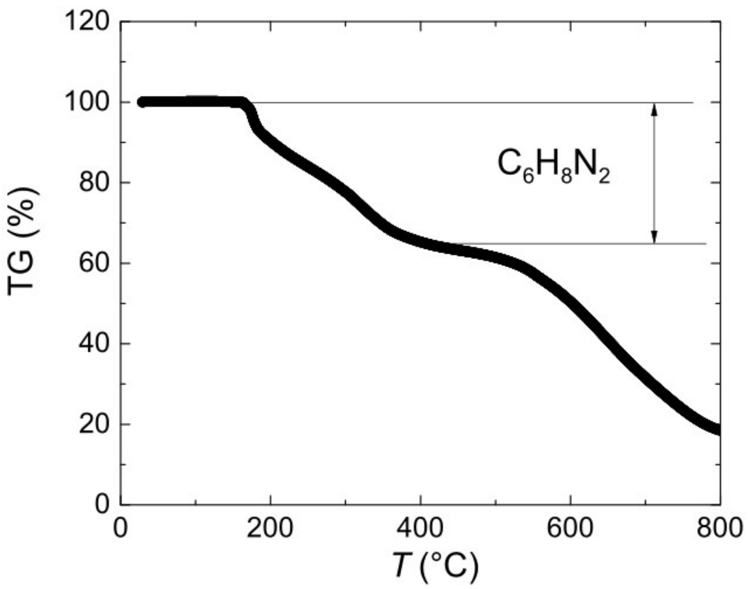

Figure S1. TGA data of **1**. The first step corresponds to the decomposition of 2,6-dimethylpyrazine organic ligand.

2. IR and Raman measurements

We performed both infrared (IR) and Raman spectroscopy measurements and the results are presented in Fig. S2. IR spectroscopy was performed using a Magna 550 FT-IR spectrometer (Nicolet Instruments, France) in the range from 400 to 2000 cm^{-1}.

Raman spectroscopy was measured by HORIBA LabRAM HR Evolution. The positions of characteristic peaks are listed in Table S1. The spectra are dominate by the vibrational mode of 2,6-dimethylpyrazine molecules. By comparison with the results of density functional theory (DFT) calculations for 2,6-dimethylpyrazine molecules, we assigned the observed peaks to the corresponding vibrations (Table S1).

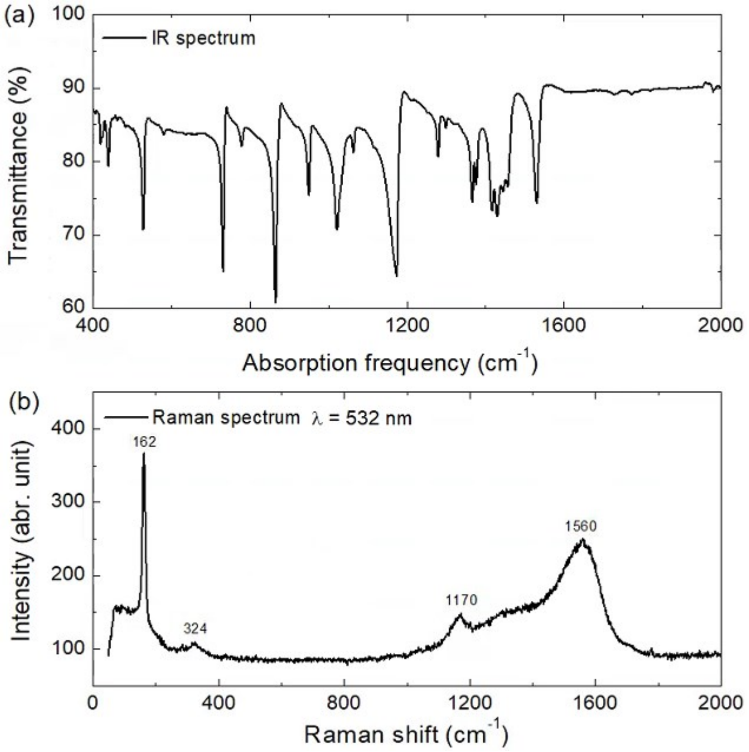

Figure S2. (a) IR and (b) Raman spectroscopy of **1**. The incident photon wavelength for Raman measurement is 532 nm. Four characteristic Raman shift peaks appear at 162, 324, 1170, and 1560 cm^{-1}.

Table S1 Observed and calculated frequencies (cm^{-1}).

IR	Raman	Assignments*
	162	Ring torsion
	324	Ring torsion
419		C-CH_3 bending
439		Ring torsion
528		C-CH_3 wagging
731		C-CH_3 wagging
865		C-H wagging
950		C-H wagging
1021		CH_3 rocking
1173	1170	C-H bending
1280		Ring stretching
1366		CH_3 bending symmetric
1430		CH_3 bending asymmetric
1530		Ring stretching

	1560	Ring stretching

* From the DFT calculations by A. Pawlukojć et al. Journal of Molecular Structure, 2008, **892,** 261–267
